# How Game Location Affects Soccer Performance: T-Pattern Analysis of Attack Actions in Home and Away Matches

**DOI:** 10.3389/fpsyg.2017.01415

**Published:** 2017-08-21

**Authors:** Barbara Diana, Valentino Zurloni, Massimiliano Elia, Cesare M. Cavalera, Gudberg K. Jonsson, M. Teresa Anguera

**Affiliations:** ^1^Human Sciences for Education Department, University of Milano-Bicocca Milan, Italy; ^2^Department of Psychology, Catholic University of the Sacred Heart Milan, Italy; ^3^Human Behavior Laboratory, University of Iceland Reykjavik, Iceland; ^4^Faculty of Psychology, University of Barcelona Barcelona, Spain

**Keywords:** analysis of observational data, T-Patterns, sport performance analysis, game location, soccer

## Abstract

The influence of game location on performance has been widely examined in sport contexts. Concerning soccer, game-location affects positively the secondary and tertiary level of performance; however, there are fewer evidences about its effect on game structure (primary level of performance). This study aimed to detect the effect of game location on a primary level of performance in soccer. In particular, the objective was to reveal the hidden structures underlying the attack actions, in both home and away matches played by a top club (Serie A 2012/2013—First Leg). The methodological approach was based on systematic observation, supported by digital recordings and T-pattern analysis. Data were analyzed with THEME 6.0 software. A quantitative analysis, with nonparametric Mann–Whitney test and descriptive statistics, was carried out to test the hypotheses. A qualitative analysis on complex patterns was performed to get in-depth information on the game structure. This study showed that game tactics were significantly different, with home matches characterized by a more structured and varied game than away matches. In particular, a higher number of different patterns, with a higher level of complexity and including more unique behaviors was detected in home matches than in the away ones. No significant differences were found in the number of events coded per game between the two conditions. THEME software, and the corresponding T-pattern detection algorithm, enhance research opportunities by going further than frequency-based analyses, making this method an effective tool in supporting sport performance analysis and training.

## Introduction

The influence of game location on performance has been investigated in sport contexts for more than 30 years. Courneya and Carron (1992) defined the so-called “home advantage” as “the term used to describe the consistent finding that home teams in sport competitions win over 50% of the games played under a balanced home and away schedule” (p. 13). Research has pointed out how athletes and teams perform significantly better when competing at home (Allen and Jones, 2014; Sarmento et al., 2014). While it is true that home advantage does not play the same role in all sports (Jones, 2013), there are no sports where athletes or teams perform better when playing away from their home venue (Courneya and Carron, 1992; Nevill and Holder, 1999; Jones et al., 2015).

Comprehensive models have been developed to guide the understanding of the home-advantage phenomenon. An outline on recent research (Allen and Jones, 2014) identified three models which take different positions on the explanation of the home advantage phenomenon: the standard model (Courneya and Carron, 1992; Carron et al., 2005), the territoriality model (Neave and Wolfson, 2003), and the “home disadvantage” model (Wallace et al., 2005).

Relevant to this work is the operationalization of the link between outcomes and performance, offered by Courneya and Carron (1992) within their standard model. We will refer to this model when speaking about three performance levels. The primary one represents the fundamental skill execution (e.g., free throw percentage in basketball, penalties per game in soccer); the secondary is the intermediate or scoring aspect of performance (e.g., points scored in basketball); and the tertiary corresponds to the traditional outcome measure (e.g., win—loss ratio).

Concerning soccer, game-location affects positively the secondary and, more often, the tertiary level of performance, representing an important factor in determining the result of a game (e.g., Pollard, 1986, 2006; Brown et al., 2002; Wolfson et al., 2005; Pollard and Gómez, 2013).

However, there are fewer evidences about the effect of game location on game structure (primary level of performance). Most of the studies analyzed professional British soccer teams. Sasaki et al. (1999) found that the 1st division team they studied from the 1996 to 1997 season performed a greater number of goal attempts, shots on target, shots blocked, shots wide, and successful crosses during home matches. Tucker et al. (2005) analyzed the matches of a professional team and found significant differences in the frequency of corner, crosses, dribbles, passes, shots (more in home matches), clearances, goal kicks, gains of possession, and losses of control (more in away matches). However, Taylor et al. (2008) showed that the outcome of most behaviors of the professional team they studied were not influenced by game location.

Other studies have tended to aggregate performance of different teams during analysis. Carmichael and Thomas (2005) revealed that in the Premier League home teams have significantly higher performance measures for attack indicators, such as shots and successful passes in the scoring zone, while away teams committed significantly more fouls and suffered more yellow and red cards. Lago-Peñas and Lago-Ballesteros (2011) analyzed 380 games of the Spanish professional soccer league, focusing on the effects of game location and team quality in determining technical and tactical performances, and showed that home team have significantly higher means for goal scored, total shots, shots on goal, attacking moves, box moves, crosses, offsides committed, assists, passes made, successful passes, dribbles made, successful dribbles, ball possession, and gains of possession, while visiting teams presented higher means for losses of possession and yellow cards. However, Seçkin and Pollard (2008) in their analysis of 301 matches during the season 2005–2006 in the Turkish Super League showed that the success rates for shots, fouls and disciplinary cards do not differ between home and away teams.

The contradictory findings showed in these studies may be due to the fact that the authors examined the effects of match location on single indicators of performance rather than on patterns of play. Recent studies in sport contexts have underlined the importance of identifying visual undetectable structural regularities in order to better assess the complex reality they refer to (Anguera et al., 2013; Zurloni et al., 2014a; Cavalera et al., 2015).

With our study, we propose an innovative data analysis technique for the analysis of performance on a primary level. It involves the detection of temporal patterns (T-patterns), to reveal hidden yet stable structures that underlie the interactive situations during matches. Therefore, detecting hidden patterns could help the coach to better predict both the performer's behavior and the opponent's one thanks to an integrated system that allows for an increased depth of analysis (Zurloni et al., 2014a).

This methodology is based on systematic observation, allowing the analysis of all the relevant information about any aspect related to any interactions linked with primary factors. The same single behaviors, which appear with the same frequency, can combine with each other to form different patterns of play; t-pattern analysis allows to detect these patterns, surpassing the results obtained by exclusively considering single indexes of behavior (such as number of crosses, or yellow cards).

Temporal pattern analysis (Magnusson, 2000) has been applied to a great number of research experiments in very different fields. Patterns have been used to describe, interpret and understand phenomena such as deceptive communication (Anolli and Zurloni, 2009; Zurloni et al., 2013, 2016; Diana et al., 2015), animal and human behavior (Casarrubea et al., 2015a,b) a wide variety of observational and sports studies, such as analysis of soccer team play (Camerino et al., 2012; Castañer et al., 2016), deception detection in doping cases (Zurloni et al., 2014b), motor skill responses in body movement and dance (Castañer et al., 2009), effectiveness of offensive plays in basketball (Remmert, 2003; Fernández et al., 2009) and futsal (Sarmento et al., 2015) or tactics employed by runners (Aragón et al., 2015).

Only few studies have recently applied T-pattern methodology to soccer. In a preliminary investigation, Jonsson et al. (2010) have examined T-pattern in five Icelandic and nine international soccer matches and showed a correlation between the number of patterns identified in each match and the coaches' ratings of team performance. Moreover, data showed a more defined temporal structure in international matches than in national ones, suggesting that international soccer matches are characterized by the presence of a more structured game. In another study, thirteen national and seven international soccer matches were coded using T-pattern analysis, confirming that the players' behavior is more synchronized than the human eye can detect and suggesting that high levels of synchrony are correlated with a good evaluation of performance by professional coaches (Jonsson et al., 2003). Camerino et al. (2012) used T-pattern analysis in order to analyze five National League (Liga) matches and five Champions' League matches from the 2000 to 2001 season of FC Barcelona. T-patterns detected revealed regularities in the playing styles of the observed team, including ball possession and ball position patterns during the attacking actions. Zurloni et al. (2014a) compared the T-patterns of attack actions in the won matches and in the lost ones played by a top club of the Italian National League Championship (Serie A) over the 2012–2013 season. The number of pattern occurrences and the number of different T-patterns detected was greater for lost matches and lower for the won matches, whereas the number of events coded was similar.

Rather than focusing on single indicators of performance, this paper aims to detect the effect of game location on the structure of play (primary level of performance) analyzing how single behaviors can combine to form patterns of play. Specifically, we will focus on attack actions, comparing T-patterns detected in home and away matches. Within the T-pattern approach, we define diversity of patterns as the number of unique behaviors included in patterns and pattern complexity as the synthesis of its length (the number of events that composes a pattern) and the number of levels (the hierarchical structure of a pattern). Given that, our hypotheses are:

H1: higher number of different patterns in home than in away matches;
H2: higher patterns' diversity in home than in away matches;
H3: higher complexity (more length and levels) of patterns in home than in away matches.

## Methods

We employed a systematic observation (Anguera, 1979; Anguera et al., 2011; Portell et al., 2015a) combined with recent technology, bringing great advantages in terms of recording quality, measurement of time, and capture of co-occurrences or diachrony (Borrie et al., 2002). We designed an *ad hoc* observation instrument, and observation was active, methodologically rigorous, non-participative, and characterized by total perceptivity. The systematic observation became more and more widespread within sport research (Lapresa et al., 2013a,b), because of its high flexibility and adaptability (Sánchez-Algarra and Anguera, 2013; Portell et al., 2015b).

The methods of analyzing performance in the game of soccer have evolved from the simple use of hand notation tracking of players' movements on scale plans of pitches to the current utilization of digital video recordings and computerized analyses.

### Design

We consider three main criteria to give a taxonomic definition of the observational design, as applied to our study (Anguera et al., 2011). It was nomothetic (as opposed to idiographic, which refers to the number of subjects observed), since we observed different matches; punctual (as opposed to continuous, referring to the number of observations conducted on the same subject); multidimensional (as opposed to unidimensional, referring to the number of dimensions/criteria, which are in correspondence with observation instrument).

Using an N/P/M (nomothetic, punctual, multidimensional) design is the reason behind decisions made regarding the structure of the observation instrument, the type of data, data quality control, and data analysis.

### Participants

We analyzed all games played by a top club during the first leg (19 matches) of the Italian National League Championship (Serie A), over the 2012–2013 season. Table 1 reports half-time and full-time results for comparison (this data was also included as mixed criteria in the coding instrument—see Section Instruments).

**Table 1:** Half-time and full-time results for the observed matches.

**Observed team home/away**	**Half-time result (observed team first)**	**Full-time result (observed team first)**	**Position in table (higher/lower than rival team)**	**Fixture**
Away	1-1	1-2 (loss)	^*^	1
Home	2-1	3-1 (win)	Lower	2
Away	0-0	2-2 (tie)	Lower	3
Home	0-1	1-2 (loss)	Lower	4
Away	1-1	3-3 (tie)	Higher	5
Home	0-0	1-0 (win)	Higher	6
Away	1-1	2-3 (loss)	Lower	7
Home	1-0	1-0 (win)	Lower	8
Away	0-2	2-3 (loss)	Higher	9
Home	0-0	1-1 (tie)	Lower	10
Home	0-1	0-2 (loss)	Lower	11
Away	0-0	0-0 (tie)	Higher	12
Home	1-1	1-1 (tie)	Lower	13
Away	1-1	3-1 (win)	Higher	14
Away	1-1	2-2 (tie)	Higher	15
Home	1-1	2-2 (tie)	Lower	16
Away	0-0	0-1 (loss)	Lower	17
Home	1-0	3-0 (win)	Higher	18
Away	2-3	3-4 (loss)	Higher	19

* *, N/A*.

This study has been approved by the Bioethic Committee of the University of Barcelona (Institutional Review Board IRB00003099).

### Instruments

#### Coding instrument

LINCE, v. 1.2.1 is a freely available software program (Gabín et al., 2012) that can be loaded [www.observesport.com and/or www.menpas.com] with purpose-designed observation instruments for the systematic recording and coding of events. The time of occurrence and duration of events (in seconds or frames) are automatically registered. The program also incorporates a data quality control tool and allows datasets and results to be exported in different formats. LINCE software operates on fixed, mixed and changing criteria of the observation, therefore making it a consistent tool for the observational design. LINCE was used to record and code each of the 19 games, and also to check the quality of the data.

#### Observation instrument

**Figure 1:**
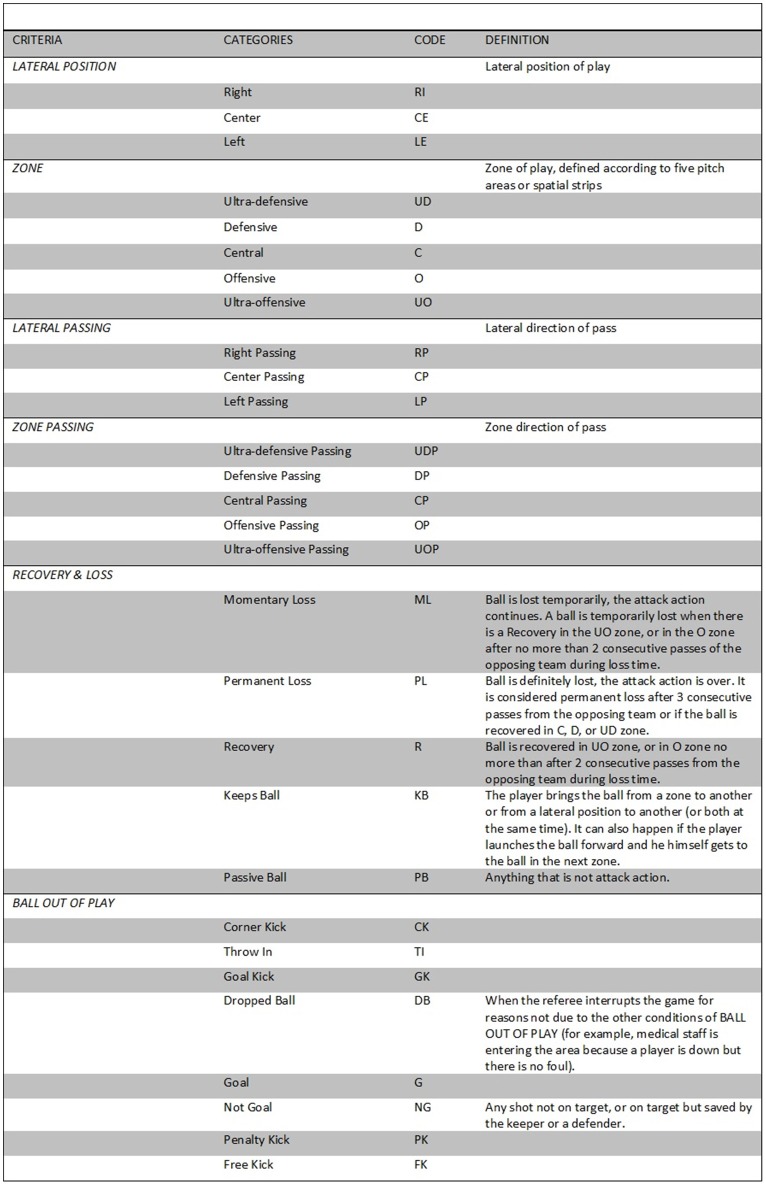
Observation instrument.

The observation instrument combines field format and category systems (Figure 1). The field format system comprised different dimensions/criteria, each of which formed the basis for an exhaustive and mutually exclusive category system. The fixed criteria are entered at the beginning of the match and are independent from the dynamic of play, while the mixed criteria apply every time there is a change in the score, number of players and between the first and the second half of the match. The changing criteria are coded throughout the whole match (i.e., passing, lateral position, shot, recovery). Each of these criteria gives rise to respective category systems that fulfill the conditions of exhaustiveness and mutual exclusivity (E/ME).

**Figure 2:**
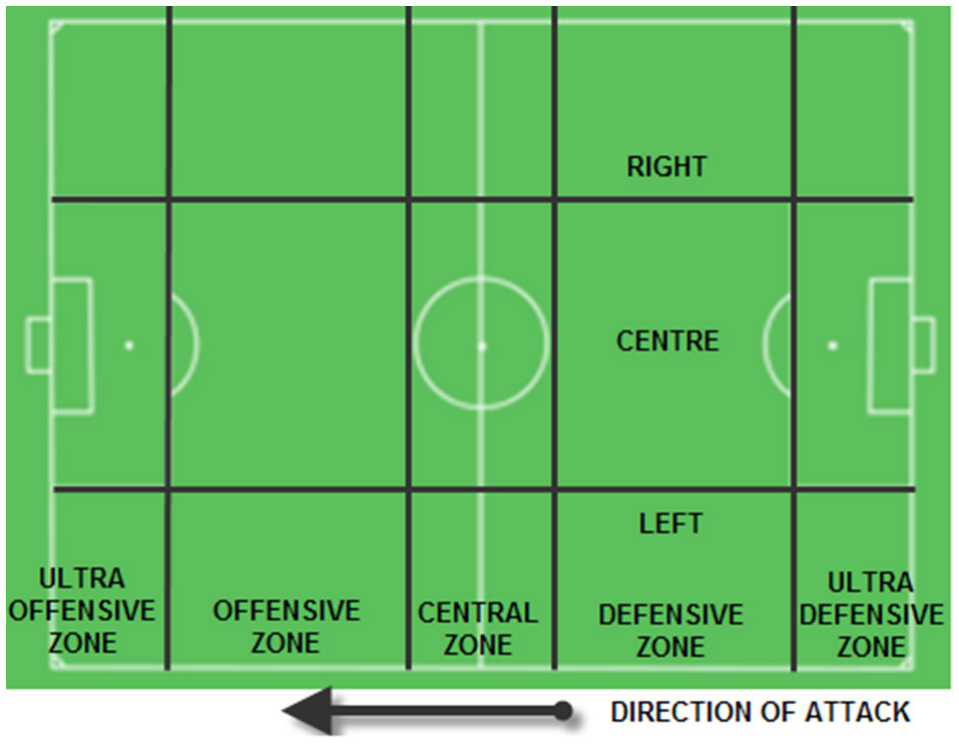
Zones and lateral positions. Adapted from Camerino et al. (2012).

The dimensions/criteria considered in the present observation instrument correspond to the following criteria: lateral position, zone (Figure 2), lateral passing, zone passing, recovery and loss, ball out of play.

### Procedure

In this study, we decided to focus on attack actions mainly because of shooting restrictions, since our video data were obtained from the TV recordings of the matches.

We defined an attack action as an action that brings the ball in the ultra-offensive zone and can end with: Goal (G), Non-Goal (NG), and Permanent Loss (PL) (see Figure 1 for a complete description of events coded); this includes penalties, corner kicks, free kicks in ultra-offensive zone, and throw-ins in the ultra-offensive zone. Each attack action was coded according to two possibilities: (1) starting from the first pass that crosses the offensive line; (2) the same was coded when the ball got to the ultra-offensive zone thanks to a free kick or a throw-in conceded to the observed team without their active effort in bringing the ball to the zone. Therefore, actions starting with a pass were not interrupted in coding by temporary pauses (such as throw-ins, free kicks, or Temporary Losses, defined as a non-possession of the ball by the observed team for no longer than 2 passes completed by the rival team) as long as the interruptions let the action proceed [e.g., during the attack action the ball is taken by a defense player, who passes it on to a different player from his team who then loses the ball to the observed team (temporary loss case)]. Permanent Loss, Goals, and Non-Goals (shots on or off-target, or deviated/saved by rival team) work as interruption lines in the attack actions coding. These interruption lines, however, do not interfere with the subsequent pattern analysis (see Section Data Analysis), which considers the coded match as a whole and will take into account time passed between events, its main value being that of allowing for similar patterns to be found when time “stretches” are insignificantly different (on the basis of pattern search parameters).

Two observers used LINCE software (Gabín et al., 2012) to code the games selected. The same software calculated Cohen's kappa coefficient (Cohen, 1960) for all the criteria, by comparing two registered data files (~5% of all the collected data) related to the same match. The values ranged between 0.75 and 0.85, which provides a satisfactory guarantee of data quality. However, when particular disagreements were identified, the specific cases were discussed and agreed on by the two coders before moving on to the complete analysis.

### Data analysis

Datasets were analyzed with THEME 6.0 (http://patternvision.com/). This software detects the temporal structure of data sets, revealing repeated patterns (T-patterns) that regularly or irregularly occur within a period of observation. A T-pattern is essentially a combination of events where the events occur in the same order, with the consecutive time distances between consecutive pattern components remaining relatively invariant, regardless of the occurrence of any unrelated event in between them (Magnusson, 2005). THEME software allows the detection of repeated temporal patterns even when multiple unrelated events occur in between components of the patterns.

A quantitative analysis, with nonparametric and descriptive statistics, was carried out to test the hypotheses. A qualitative analysis on more complex patterns was performed to get in-depth information on the game structure expressed by the team. We chose to consider the more complex patterns because they represent the highest level of organization expressed by the team in the two conditions.

## Results

Of the 19 matches played, the observed team collected 30 points (3 points per victory, 1 per tie, and 0 for losses), with 9 wins, 7 losses, and 3 ties. Six wins out of 9 were obtained in home matches, highlighting a home advantage for the observed team at tertiary level of performance (Courneya and Carron, 1992).

### Comparison between home and away matches

We performed descriptive statistics as a preliminary analysis. Extreme values identified 5 matches as potential outliers. We decided to exclude them from next analysis. According to sample size and distribution, we used the non-parametrical Mann–Whitney test to assess differences between home and away matches. Here follow the results (Table 2) for each parameter considered in our hypotheses: a higher number of different patterns was detected in home matches (*Mdn* = 127) than in the away ones (*Mdn* = 42), *U* = 1, *Z* = 3.003, *p* = 0.001, *r* = 0.80. Home matches included more unique behaviors in their patterns (*Mdn* = 31) than away matches (*Mdn* = 20), *U* = 2.5, *Z* = 2.83, *p* = 0.002, *r* = 0.76. Home matches' patterns were more complex, with a higher number of levels (*Mdn* = 1.9817) than the away ones (*Mdn* = 1,381), *U* = 2, *Z* = 2.875, *p* = 0.002, *r* = 0.77, and a higher length (*Mdn* = 3.2557) than the away ones (*Mdn* = 2.4048), *U* = 2, *Z* = 2.875, *p* = 0.002, *r* = 0.77. No significant differences were found in the number of events coded per game between the home (*Mdn* = 87) and the away condition (*Mdn* = 75), *p* = 0.259.

**Table 2:** Results recap.

	**Home median**	**Away median**	***U***	***Z***	***p***	***r***
Unique patterns	127	42	1	3.003	*0.001*	*0.80*
Unique behaviors	31	20	2.5	2.83	*0.002*	*0.76*
Patterns' levels	1.9817	1.381	2	2.875	*0.002*	*0.77*
Patterns' length	3.2557	2.4048	2	2.875	*0.002*	*0.77*
Number of events coded per game	87	75	NS	NS	*0*.259	NS

*P- and r-values in italics are considered significant and related to a medium-large effect size*.

A second analysis was performed to qualitatively compare home and away attack strategies, by combining the single datasets, from each of the two conditions, for the T-pattern detection. THEME detected 721 different T-patterns occurring in at least 80% of the home matches (*p* = 0.005). Two hundred and fifty-six out of 721 different T-patterns (36%) have four or more events (minimum threshold when speaking about complexity, since it means considering patterns that connect at least two different sub-patterns).

**Figure 3:**
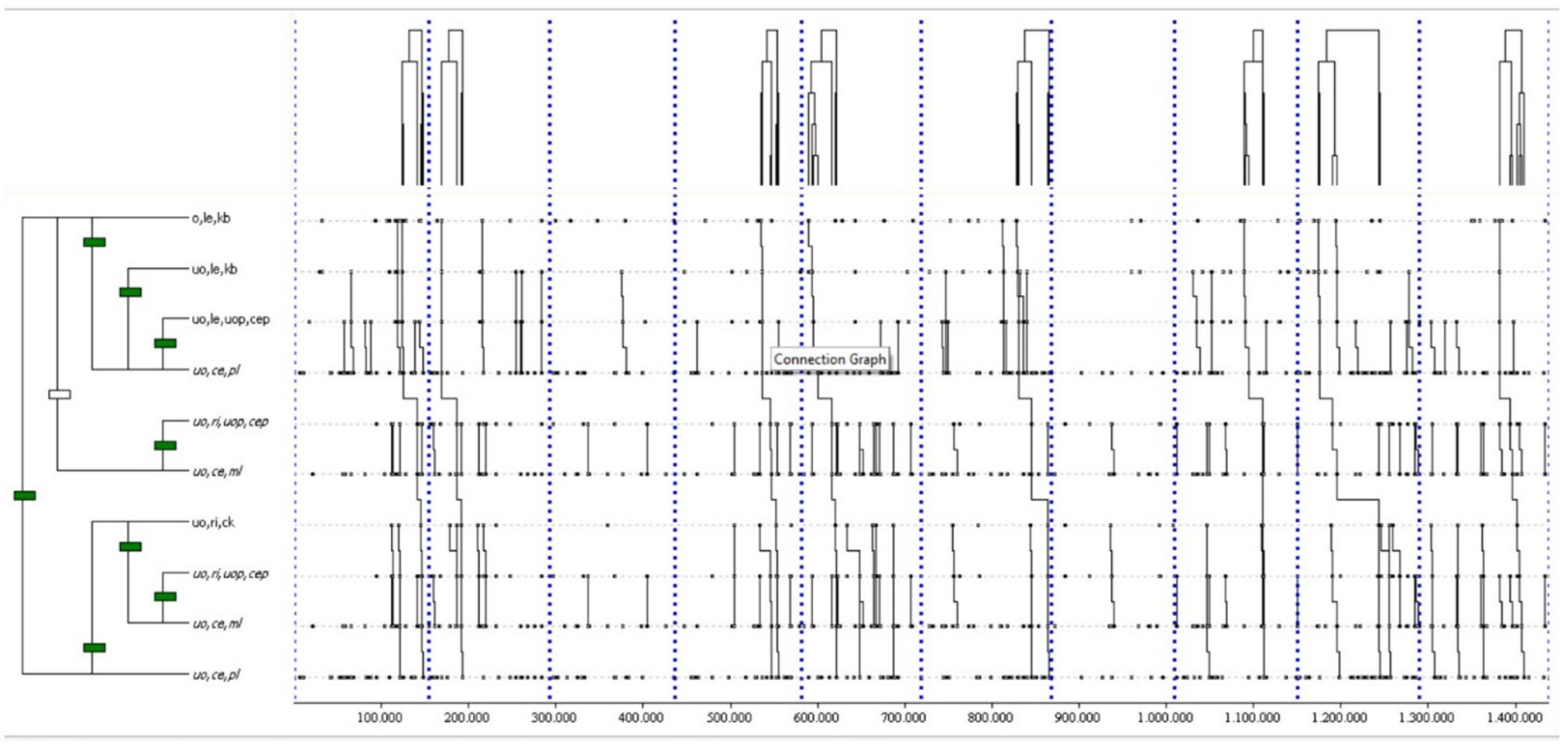
The most complex T-pattern from home matches. It occurred in 80% of the games analyzed. Events are (1) o,le,kb; (2) uo,le,kb; (3) uo,le,uop,cep; (4) uo,ce,pl; (5) uo,ri,uop,cep; (6) uo,ce,ml; (7) uo,ri,ck; (8) uo,ri,uop,cep; (9) uo,ce,ml; and (10) uo,ce,pl.

The most complex T-pattern (see Figure 3,^1^) was detected 8 times in at least 80% of home matches, with a length of 10 events and 5 levels. It shows a ball keeping in left offensive area (o,le,kb), another ball keeping in left ultra-offensive area (uo,le,kb), a pass from the left ultra-offensive area to the central ultra-offensive area (uo,le,uop,cep), a permanent loss in this area (uo,ce,pl), a pass from the right ultra-offensive area to the central ultra-offensive area (uo,ri,uop,cep), a momentary loss in this area (uo,ce,ml), a corner kick from the right ultra-offensive area (uo,ri,ck), another pass from the right ultra-offensive area to the central ultra-offensive area (uo,ri,uop,cep), another momentary loss in this area (uo,ce,ml) and finally a permanent loss in this area (uo,ce,pl).

THEME detected 203 different T-patterns occurring in at least 80% of the away matches (*p* = 0.005). Twenty-nine out of 203 different T-patterns (14%) have four or more events.

**Figure 4:**
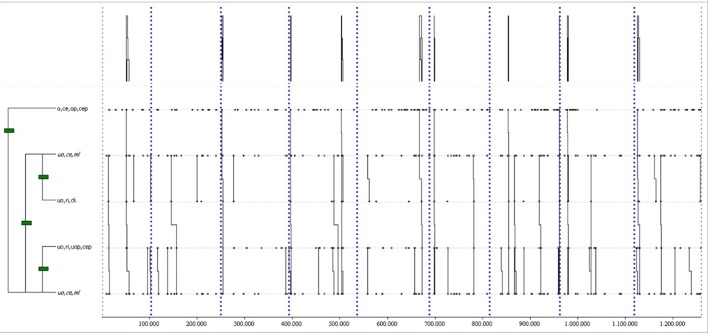
The most complex T-pattern from away matches. It occurred in over 80% of the games analyzed. Events are (1) o,ce,op,cep; (2) uo,ce,ml; (3) uo,ri,ck; (4) uo,ri,uop,cep; and (5) uo,ce,ml.

The most complex T-pattern (see Figure 4) was detected 10 times, with a length of 5 events and 3 levels. It shows a pass from the central offensive area to the same area (o,ce,op,cep), followed by a momentary loss in the central ultra-offensive area (uo,ce,ml), a corner kick from the right ultra-offensive area (uo,ri,ck), a pass from the right ultra-offensive area to the central ultra-offensive area (uo,ri,uop,cep), and again a momentary loss in this area (uo,ce,ml).

### Comparing positive attack actions between home and away matches

In order to explore the efficacy of the attack actions both in home and in away matches, we considered for a deeper analysis only those t-patterns that involved *Goals* or *NoGoals* (shots).

**Figure 5:**
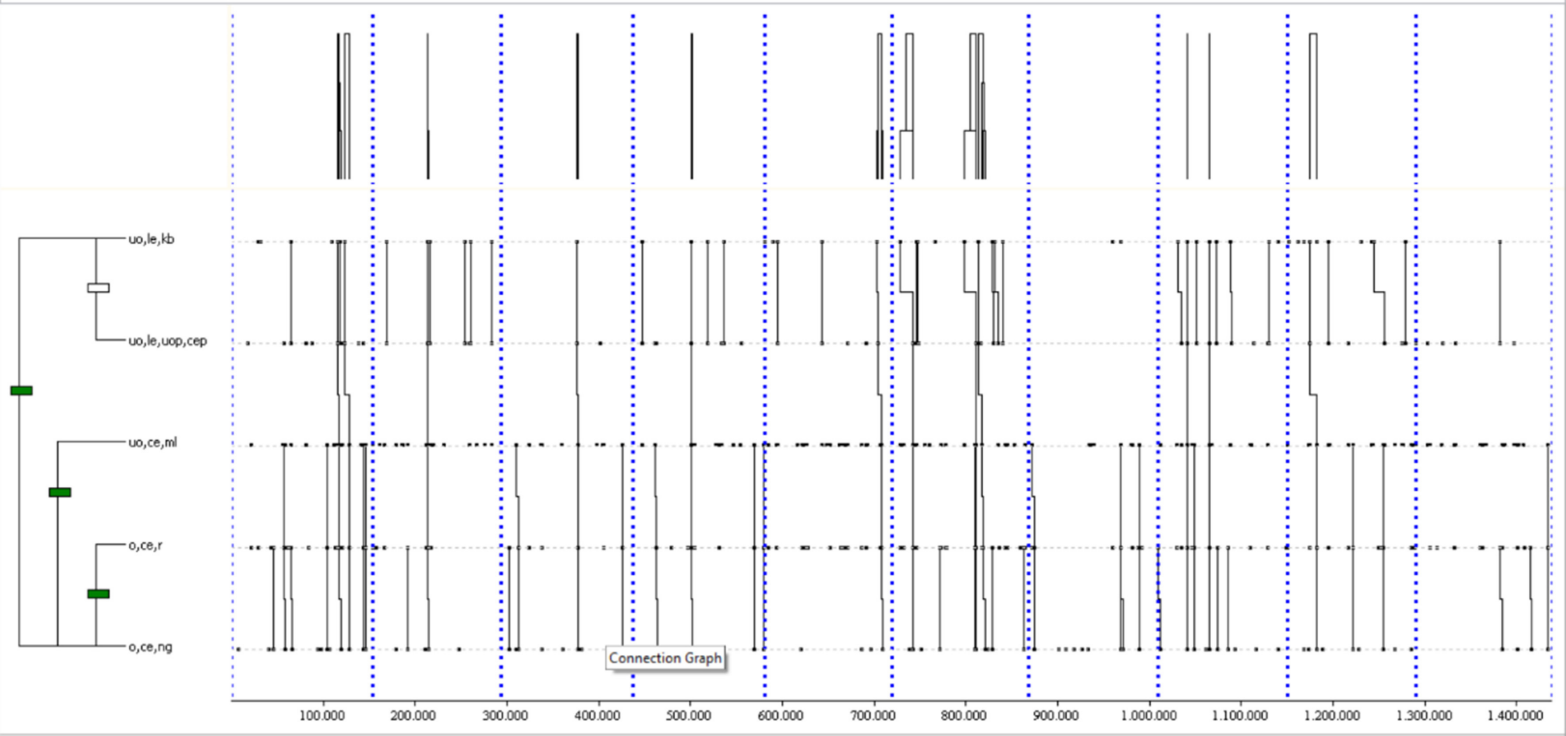
One of the most complex T-patterns from home matches with a NoGoal. It occurred in 80% of the games analyzed. Events are (1) uo,le,kb; (2) uo,le,uop,cep; (3) uo,ce,ml; (4) o,ce,r; and (5) o,ce,ng.

In home matches, THEME detected 82 common T-patterns including at least a Goal or a NoGoal event (*p* = 0.005). The most complex T-pattern (see Figure 5) was detected 12 times in at least 80% of home matches, with a length of 5 events and 3 levels. It shows a ball keeping in the left ultra-offensive area (uo,le,kb), followed by a pass from that zone to the central ultra-offensive area (uo,le,uop,cep), a momentary loss in this area (uo,ce,ml), a recovery in the central offensive area (o,ce,r), and finally a NoGoal (shot) from the same area (o,ce,ng).

**Figure 6:**
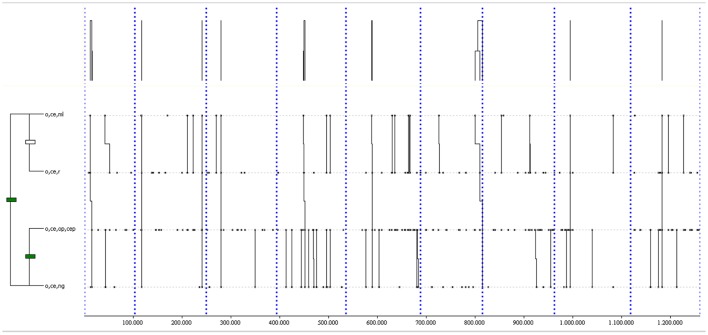
The most complex T-pattern from away matches with a NoGoal. It occurred in over 80% of the games analyzed. Events are (1) o,ce,ml; (2) o,ce,r; (3) o,ce,op,cep; and (4) o,ce,ng.

THEME detected 20 common T-patterns in away matches with at least a Goal or a NoGoal (*p* = 0.005). The most complex T-pattern (see Figure 6) was detected 9 times in at least 80% of away matches, with a length of 4 events and 2 levels. It describes a momentary loss in the central offensive area (o,ce,ml), followed by a recovery in the same area (o,ce,r), a pass from the central offensive area to the same area (o,ce,op,cep), and a NoGoal from the same area (o,ce,ng).

## Discussion

Since the average number of events coded per game does not significantly differ between home and away matches, we can affirm that differences in patterns between the two conditions are not due to the number of events coded.

Mann–Whitney test results allow us to refuse the null hypothesis, hence confirming home and away matches to be significantly different in terms of patterns' number, diversity, and complexity. Descriptive statistics show that in home matches THEME detected a greater number of different patterns (H1 confirmed), characterized by a higher diversity (H2 confirmed) and complexity (H3 confirmed). Moreover, according to Cohen's classification (Cohen, 1988), results showed a large effect size, ranging from 0.7 to 0.8.

In terms of primary level of performance, home matches are characterized by a more structured (higher number of levels in patterns) and varied (longer patterns, each composed by different events) game; this could be due to the team having more confidence when applying rehearsed tactics, as well as new ones. In fact, away matches present a more stereotyped game, with simpler patterns; there seem to be more difficulties in structuring and changing the game tactics. The greater number of different behaviors, identified in home matches' patterns, confirms these data and provides the team with a wider range of opportunities not to be predictable, making it harder for the opponents to respond appropriately.

Qualitatively analyzing the more complex patterns of the two conditions, we noticed that they end similarly, with a corner kick and ball in the ultra-offensive area (positive events speaking about attack actions in soccer, because they can lead to a chance on goal). However, there is a difference if we focus on the events preceding this outcome. It seems that, in home matches, ball possession and the widening of the play on the lateral sides are an important part of the team's strategy. There are numerous and continuous changes of play with crosses from the sides directed to the central ultra-offensive area but there seem to be difficulties in exploiting those crosses, mistakes, and adversaries' defenses tend to prevail. Regarding away matches, the limited number of events composing the pattern makes it difficult to draw general conclusions about the team strategy, apart from highlighting a difficulty in the central penetration (from the offensive central to the ultra-offensive central zone), probably linked to a difficulty in widening the play. This simplicity, however, is an index of a difficulty for the team in creating and establishing a functional game strategy to getting important attack chances, such as the one described in the pattern. You could also say that this kind of occasions happened more randomly in away matches compared to what happened in the home condition.

By restricting the analysis to positive attack actions, we found that the number of patterns including goals and shots to goal is much higher in home rather than in away matches (on a 4 to 1 ratio). This partially confirms previous findings. Sasaki et al. (1999), for example, found that the team they studied performed a greater number of goal attempts and shots on target during home matches. Different studies have shown significantly higher performance measures for attack indicators, such as shots to goal (Carmichael and Thomas, 2005; Tucker et al., 2005) and goals scored (Lago-Peñas and Lago-Ballesteros, 2011) when the teams they observed were performing at home. It seems that, in home matches, the shot to goal is more probably the result of a strategically repeated structured maneuver, compared to what happened in the away condition.

Qualitative analysis of the more complex patterns reveals that, like the previous case, they end similarly in the two conditions (a ball recovery in the offensive central zone and a shot from outside). However, while in home matches these events are preceded by a ball possession on the lateral sides and a penetration in the ultra-offensive zone, in the away matches the game develops in the central offensive zone, an area where it is more difficult for the team to create scoring chances.

## Conclusion and practical applications

An important limitation of this study, similar to other studies previously discussed (e.g., Sasaki et al., 1999; Tucker et al., 2005; Taylor et al., 2008) is that we considered a single's team performance over a sustained period. Even if tactics and strategies are unique to individual teams and what is successful for one team may therefore not be for another (Tucker et al., 2005), aggregating performance of different teams during analysis may favor generalization of findings. Moreover, we did not examine the effects of match location on technical and tactical performances as a function of team quality. Several studies have revealed that team quality affects the degree of home advantage obtained in sport (i.e., Schwartz and Barsky, 1977; Madrigal and James, 1999; Lago-Peñas, 2009; Lago-Peñas and Lago-Ballesteros, 2011).

However, in line with the findings of other studies that have applied T-pattern methodology to soccer (Jonsson et al., 2003, 2010; Camerino et al., 2012; Zurloni et al., 2014a), the results obtained and discussed above strengthen the belief that T-pattern analysis is an effective tool, producing different potential ways of supporting research in sport performance analysis and more specifically in game location effects.

In general, this study shows that game tactics are different between home and away matches. The home advantage detected at a tertiary level of performance is linked to a difference in the primary level, that t-pattern analysis translated in a more structured and varied game (more different patterns, longer and more complex), in which the ball possession in midfield and the numerous attempts to widen the play on the sides seem to represent an important part of the team's strategy. The situation is different for away matches; the “poor and stereotyped” structure of the detected patterns does not allow us to draw general indications on game attack strategies, apart from a difficulty in creating and establishing stable structures of play. It would be probably useful for the team to replicate the same “home strategy” of widening of the play, given the inefficacy of central penetration, which often leads to shots from outside.

These data need to be further developed and analyzed, for example by increasing the sample size, comparing tactics between different teams and extending the analysis to defensive play as well (*ad hoc* footage would be needed, since television footage usually follows the ball's movements).

These results also point toward the need to investigate the potential link between temporal structure detection and soccer managers' observations. In fact, the chance to create an observation instrument tailored on the needs of a specific team, exploiting the collaboration of the manager and his technical staff, would achieve even more significant results.

THEME and the corresponding T-pattern algorithm offer alternative means of analyzing team performance at a primary level, contributing to the research on the effects of game location, enriching the models already existing in literature and creating a different perspective that can be used to increase the team's performance at all levels. An important step will be to support this kind of analysis with the measurement of psycho-physiological factors, to understand which and how specific state and trait variables influence performance, aiming at a better monitoring and training of the team as a whole.

## Author contributions

BD and VZ: Study designing, method development, data analysis, paper writing. ME: Data acquisition and coding, data analysis, paper writing. CC: Study designing, data acquisition and coding, paper writing. GJ: Method development, data analysis. MA: Method development, paper writing. All authors made suggestions and critical reviews to the initial draft and contributed to its improvement (until reaching the final manuscript), which was read and approved by all authors.

### Conflict of interest statement

The authors declare that the research was conducted in the absence of any commercial or financial relationships that could be construed as a potential conflict of interest.

## Footnotes

^1^How to read the pattern tree graph: the left box of Figures 3–6 shows the events occurring within the pattern, listed in the order in which they occur within the pattern. The first event in the pattern appears at the top and the last at the bottom. The lower right box shows the frequency of events within the pattern, each dot means that an event has been coded. The pattern diagram (the lines connecting the dots) shows the connection between events. The number of pattern diagrams illustrates how often the pattern occurs. Sub-patterns also occur when some of the events within the pattern occur without the whole of the pattern occurring. The upper box illustrates the real-time of the pattern. The lines show the connections between events, when they take place and how much time passes between each event.
